# Alpha-lipoic acid improves the quality of ram spermatozoa stored at 4°C by reducing oxidative stress and increasing mitochondrial potential

**DOI:** 10.3389/fvets.2023.1345016

**Published:** 2024-01-08

**Authors:** Xiaomei Sun, Liuming Zhang, Yan Kang, Xuyang Wang, Caiyu Jiang, Jian Wang, Tariq Sohail, Yongjun Li

**Affiliations:** Key Laboratory for Animal Genetics & Molecular Breeding of Jiangsu Province; College of Animal Science and Technology, Yangzhou University, Yangzhou, China

**Keywords:** alpha-lipoic acid, sheep, spermatozoa, oxidative stress, low temperature

## Abstract

**Introduction:**

Ram spermatozoa inevitably produce a large number of reactive oxygen species (ROS) during liquid storage, leading to oxidative stress and a decline of spermatozoa quality. Therefore, it is particularly important to add exogenous antioxidants during the process of semen liquid preservation. The purpose of this study is to investigate whether adding alpha-lipoic acid (ALA) to ram semen can reduce oxidative stress and enhance spermatozoa quality during the liquid storage at 4°C.

**Methods:**

Different concentrations of ALA (0, 0.025, 0.05, 0.1, 0.5, 1 mM) were added to semen and stored at 4°C. During storage at 4°C, spermatozoa motility, kinetic parameters, membrane integrity, acrosome integrity, energy metabolism parameters (mitochondrial membrane potential (ΔΨM) and adenosine triphosphate (ATP)) and oxidative stress parameters [ROS, malondialdehyde (MDA), total antioxidant capacity (TAC), superoxide dismutase (SOD)] were assessed.

**Results and discussion:**

The results indicated that 0.1 mM ALA significantly (*p*<0.05) improved spermatozoa total motility (TM) and progressive motility (PM), plasma membrane integrity, acrosome integrity, ΔΨM, ATP, TAC, and SOD, while significantly (*p*<0.05) reducing spermatozoa ROS and MDA content compared to the control group. In conclusion, ALA can reduce damage caused by oxidative stress in spermatozoa and effectively improve the quality of semen preserved at 4°C. And the optimal concentration is 0.1 mM.

## 1 Introduction

Hu sheep is a world-famous breed known for its high productivity (1). Compared with specialized mutton sheep breeds, the excellent characteristics of Hu sheep, such as high fertility and multiple births, stand out in large-scale house feeding (2). Artificial insemination (AI) plays an important role in the reproductive process, and the collection, processing, and preservation of semen are important factors that affect the efficiency of AI (3). In addition, low-temperature preservation involves diluting semen, slowly cooling it, and then storing it at 4°C. It induces dormancy in spermatozoa and reduces metabolic activity, thereby prolonging the lifespan of spermatozoa (4).

Oxidative stress is an important factor that impacts semen quality during the process of *in vitro* semen preservation. Semen possesses an antioxidant system that is safeguarded by various antioxidant enzymes, which help prevent oxidative stress resulting from an excess of reactive oxygen species (ROS) in the spermatozoa (5). And spermatozoa can produce a small amount of ROS, which plays an important role in spermatozoa capacitation and acrosome reaction (6, 7). However, as the duration of semen storage increases, abnormal and dead spermatozoa can generate significant amounts of ROS, leading to an imbalance in ROS level and subsequent oxidative stress (8–10). On the one hand, excessive ROS will also attack polyunsaturated fatty acids (PUFAs) in the spermatozoa plasma membrane, leading to the integrity of the spermatozoa membrane structure and causing lipid peroxidation reaction (LPO) in the spermatozoa plasma membrane (11). On the other hand, ROS can damage spermatozoa DNA by breaking DNA double strands and inducing apoptosis (12, 13). Therefore, to reduce the negative impact of oxidative stress on semen quality, antioxidants are usually added to the diluent (14). The primary mechanisms by which antioxidants exert their effects include directly removing the ROS produced, inhibiting the production of spermatozoa ROS, and removing or repairing damage caused by ROS (15).

Alpha-lipoic acid (ALA) is a vitamin-like biological non-enzymatic antioxidant with high efficiency (16, 17). ALA is a mitochondrial coenzyme synthesized from mitochondrial octanoic acid through a mitochondrial enzymatic reaction and it plays a role in the tricarboxylic acid cycle (18). Moreover, it catalyzes the generation and transfer of acyl groups in the oxidative decarboxylation process of the pyruvate dehydrogenase and α-ketoglutarate dehydrogenase complex, and plays an antioxidant and metabolic role at the cellular level (19). It is reported that ALA can play an active role in the treatment of diseases related to the human reproductive system (20, 21). Furthermore, oral administration of ALA has been shown to enhance sperm quality in both rats (22) and humans (23). However, the preservation effect of ALA at 4°C on Hu ram spermatozoa has not been demonstrated in any research. The aim of this study is to investigate whether ALA can enhance the quality of Hu ram semen preservation at 4°C by reducing oxidative stress.

## 2 Materials and method

### 2.1 Animals and semen collection

The ejaculates of five proven fertile Hu rams were collected using an artificial vagina according to procedures approved by the Animal Ethics Committee of Yangzhou University (SYXK[Su]2017–0044) and Hu sheep are known to be in estrus all year round. The study selected healthy rams aged 2 to 4 years, free of parasites. The rams were free to drink water and ingest licking bricks rich in minerals. The ram was fed 0.4 kg concentrate and 0.2 kg alfalfa every day. A total of 90 ejaculates (three times a week for each ram) were used for the experiments and the experiments were repeated five times. Ejaculates were collected from October to December 2022. The computer-assisted sperm analyzer (CASA) was utilized to evaluate the collected spermatozoa. Semen with spermatozoa motility greater than 80%, abnormal rate less than 15%, semen volume between 0.6 ~ 1.3 mL and the spermatozoa concentration between 2 × 10^9^ ~ 2.5 × 10^9^ was selected. The qualified semen was evenly mixed to eliminate individual differences.

### 2.2 Semen extender and evaluation

The 500 mL basic extender was composed of 2.5 g fructose, 12 g sodium citrate, 0.5 g Soy Lecithin, 250,000 IU penicillin sodium and streptomycin sulfate (24). ALA was dissolved in water and should be prepared in the dark. ALA (Purity>99%, Beyotime, Shanghai, China) was added to the basic extender at concentrations of 0.025, 0.05, 0.1, 0.5, and 1.0 mM, while the control was the basic extender without ALA (The concentration range of ALA was determined by a pre-experiment.). All the samples were diluted to 2×10^8^ sperm/ml using an extender containing different concentrations of ALA. The semen samples were stored at 4°C in a refrigerator. It is recorded as 0 d when the sample is placed at 4°C and it is recorded as 1 d after 24 h.

Spermatozoa motility parameters, functional integrity parameters (including plasma membrane and acrosome integrity), oxidative stress parameters [ROS, malondialdehyde (MDA), superoxide dismutase (SOD), total antioxidant capacity (TAC)], and energy metabolism-related parameters [mitochondrial membrane potential (ΔΨM), adenosine triphosphate (ATP)] were assessed on the first, third, and fifth day of semen preservation at 4°C.

### 2.3 Spermatozoa motility parameters

Parameters such as Total motility (TM, %), Progressive motility (PM, %), Straight line velocity (VSL, μm/s), Curvilinear velocity (VCL, μm/s), Average path velocity (VAP, μm/s), and Amplitude of lateral head displacement (ALH, μm) were assessed using CASA (Instrument number: ML-608JZ II, Mailang, Nanning, China). The CASA software recorded data at 30 frames per second. Samples diluted with the basic extender were incubated at 37°C for 4 min. A total of 1.4 μL semen sample was placed on a MACRO sperm counting chamber (YA-1, Yucheng, Nanjing, China) and examined at 37°C using a phase-contrast microscope (ML-800, Mailang, Nanning, China) at a magnification of 100×, equipped with a CCD-camera (MD06200C, Mailang, Nanning, China).

### 2.4 Spermatozoa functional integrity parameters

The integrity of the spermatozoa plasma membrane was assessed using the hypotonic swelling test (HOST). Semen samples preserved at 4°C were mixed with a hypotonic solution (consisting of 0.245 g sodium citrate and 0.45 g fructose dissolved in 50 mL distilled water) in a 1: 10 ratio. The osmotic pressure of the hypotonic solution was 108 m Osm/L. The evenly mixed samples were incubated at 37°C for 30 min. After incubation, 1.4 μL of the mixture was dispensed onto a specialized plate for spermatozoa counting. The coiling rate of the spermatozoa was examined using a phase-contrast microscope (CX31, Olympus Corporation, Tokyo, Japan) at a magnification of 400×, until 200 spermatozoa had been counted.

Giemsa staining was utilized to assess the integrity of the spermatozoa acrosome. Smears were taken from 10 μL of semen samples stored at 4°C. After 5 ~ 10 min, allow it to air dry naturally, and then fix it with 4% paraformaldehyde. After that, it was soaked in Giemsa stain for 3 h. After air-drying, the spermatozoa were examined under a phase-contrast microscope (CX31, Olympus Corporation, Tokyo, Japan) at a magnification of 400×, until 200 spermatozoa had been counted.

**Figure 1:**
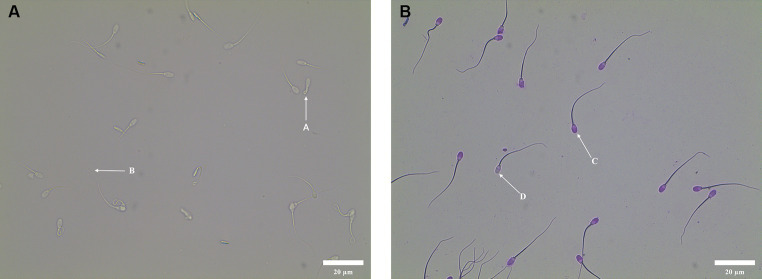
Sperm functional integrity test. **(A)** Morphology of curly tail of sperm in HOST. A: Sperm with intact membrane; B: Sperm with damaged membrane. **(B)** Acrosome morphology of sperm stained with Giemsa. D: Sperm with damaged acrosome; C: Sperm with intact acrosome.

The result of HOST incubation was shown in Figure 1A. There were two types of sperm tail: A and B, in which the tail curl type A represented intact membrane sperm, and the tail non-curl type B represented sperm with damaged membrane. The result of Giemsa staining was shown in Figure 1B. There were two types of sperm head: C and D. If the sperm head was unstained, then the acrosome was not intact (D). If the sperm head was evenly purplish red, then the acrosome was intact (C).

### 2.5 Spermatozoa oxidative stress parameters

ROS generation was measured using an ROS Assay Kit (Beyotime Institute of Biotechnology, Shanghai, China) following the manufacturer’s instructions. Briefly, the ROS content was measured by incubating spermatozoa with 300 μL DCFH-DC solution for 30 min at 37°C in dark conditions, followed by three rounds of centrifugation and washing in PBS. The samples were analyzed using a multifunctional microplate reader (488 nm excitation and 525 nm emission for DCF) and the ROS content was determined based on the fluorescence intensity.

Measurement of MDA generation was conducted using an MDA Assay Kit (Beyotime Institute of Biotechnology, Shanghai, China) following the manufacturer’s instructions. Briefly, the MDA content was measured by incubating spermatozoa with 200 μL MDA working solution for 15 min at 100°C, followed by a single centrifugation at room temperature. The samples were evaluated using a multifunctional microplate reader (absorbance at 532 nm) and the MDA content was determined based on the standard curve.

SOD activity was measured using a Total Superoxide Dismutase Assay Kit with WST-8 (Beyotime Institute of Biotechnology, Shanghai, China) following the manufacturer’s instructions. Briefly, the semen samples were lysed using the lysis buffer in the kit, followed by measuring the protein concentration using a Detergent Compatible Bradford Protein Assay Kit (Beyotime Institute of Biotechnology, Shanghai, China). The SOD activity was measured by incubating the spermatozoa with a series of reagents at 37°C for 30 min. The samples were evaluated using a multifunctional microplate reader (absorbance at 450 nm) and the SOD activity was calculated using the instruction formula.

TAC was measured using a TAC Assay Kit (Nanjing Jiancheng Bioengineering Institute, China) by measuring the absorbance of 2,2′-Azinobis-(3-ethylbenzthiazoline-6-sulphonate)^+^ (ABTS^+^) following the manufacturer’s instructions. Briefly, the samples were centrifuged, and the supernatant was collected. A series of reagents was added to a 96-well plate to quantify TAC. The samples were evaluated using a multifunctional microplate reader (ABTS^+^ absorbance at 405 nm) and the TAC was determined based on the standard curve.

### 2.6 Spermatozoa energy metabolism parameters

Mitochondrial membrane potential (ΔΨM) was measured using a ΔΨM Assay Kit with JC-1 (Beyotime Institute of Biotechnology, Shanghai, China) following the manufacturer’s instructions. The formation of JC-1 aggregates can produce red fluorescence, indicating high ΔΨM. JC-1 is a monomer that can produce green fluorescence, indicating a lower ΔΨM. Briefly, the samples were centrifuged, and the supernatant was removed. Added 500 μL JC-1 working solution to the samples and incubated them for 20 min in the dark. The samples were evaluated using a multifunctional microplate reader with an excitation wavelength of 488 nm and an emission wavelength of 525 nm for JC-1 monomer and an excitation wavelength of 525 nm and an emission wavelength of 590 nm for JC-1 aggregates. The formula provided in the instructions was used to calculate the ΔΨM.

ATP content was measured using an ATP Assay Kit (Solarbio, Beijing, China) following the manufacturer’s instructions. Briefly, the samples were centrifuged at 1000 × g for 10 min to obtain the supernatant. Add 1 mL extractive solution to 100 μL supernatant, mix well, and centrifuge at 10000 × g for 10 min to obtain the supernatant. Add 500 μL chloroform to the supernatant, mix well, and centrifuge at 10000 × g for 3 min to obtain the supernatant. Add a series of reagents to the supernatant and use a multifunctional microplate reader to measure the absorbance at 340 nm at 10 s and 3 min 10 s. The ATP content can be obtained using the formula.

### 2.7 Statistical analysis

The Shapiro–Wilk test was used to analyze the normality of the data (SPSS 25.0 software). After testing, the data showed a normal distribution. The parameters were evaluated and analyzed using a two-way repeated measures ANOVA. *p* < 0.05 means a significant difference. All results were expressed as “Mean ± SEM.”

## 3 Result

### 3.1 Effects of ALA supplementation on spermatozoa TM and PM

As shown in Table 1, the TM and PM of all ALA groups were significantly higher (*p* < 0.05) than those of the control group from day 1 to 5. The TM of the 0.1 mM group was significantly higher (*p* < 0.05) than that of the 0 mM, 0.025 mM and 0.5 mM groups on the third day. On the fifth day, the TM of the 0.1 mM group was significantly higher (*p* < 0.05) than that of the 0 mM, 0.025 mM, 0.5 mM and 1 mM groups. On the third day, the PM of 0.05 mM, 0.1 mM and 0.5 mM groups were significantly higher (*p* < 0.05) than in the other groups. The PM of the 0.1 mM group was significantly higher (*p* < 0.05) than in the 0 mM, 0.025 mM and 1 mM groups on the fifth day.

**Table 1:** Effect of different concentrations of ALA on spermatozoa TM and PM.

Parameter	Time	0 mM	0.025 mM	0.05 mM	0.1 mM	0.5 mM	1 mM
TM (%)	1 d	82.18 ± 0.29^Ac^	87.43 ± 0.67^Aab^	88.31 ± 0.39^Aa^	88.54 ± 0.37^Aa^	87.75 ± 0.58^Aab^	86.51 ± 0.65^Ab^
	3 d	76.87 ± 1.48^Bc^	80.41 ± 0.40^Bb^	85.93 ± 0.38^Aa^	85.89 ± 0.47^Ba^	82.11 ± 0.50^Bb^	85.28 ± 0.12^Aa^
	5 d	55.26 ± 0.28^Cd^	66.16 ± 2.16^Cc^	76.31 ± 1.19^Ba^	78.31 ± 0.21^Ca^	72.49 ± 0.61^Cb^	71.44 ± 0.79^Bb^
PM (%)	1 d	75.29 ± 0.60^Ab^	81.28 ± 1.78^Aa^	81.28 ± 0.72^Aa^	81.42 ± 0.69^Aa^	81.92 ± 0.96^Aa^	81.54 ± 0.34^Aa^
	3 d	68.97 ± 0.58^Bd^	71.60 ± 0.26^Bc^	78.30 ± 0.44^Aa^	79.14 ± 0.47^Ba^	78.48 ± 0.73^Aa^	75.08 ± 1.63^Bb^
	5 d	46.60 ± 0.79^Cd^	57.44 ± 1.50^Cc^	65.59 ± 1.69^Bab^	68.20 ± 0.72^Ca^	65.12 ± 2.04^Bab^	61.57 ± 0.96^Cbc^

Different superscripts (lowercase) in the same row show significant differences (*p* < 0.05). Different superscripts (uppercase) in the same column show significant differences (*p* < 0.05).

### 3.2 Effects of ALA supplementation on spermatozoa kinetic parameters

As shown in Table 2, the VSL of the 0.1 mM group was higher (*p* < 0.05) than that of the 0 mM, 0.025 mM, 0.5 mM and 1 mM groups from day 1 to 5. On the first day, the VSL of all ALA groups was higher (*p* < 0.05) than that of the control group. The 0.1 mM group was higher (*p* < 0.05) compared to the 0 mM, 0.025 mM, 0.5 mM and 1 mM groups on the first day, but it did not show a significant difference (*p* > 0.05) compared to the 0.05 mM group. On the third day, the VSL of the 0.1 mM group was significantly higher (*p* < 0.05) than that of the control, 0.025 mM, 0.5 mM and 1 mM groups, but it was not significantly higher (*p* > 0.05) than the 0.05 mM group. On the fifth day, the VSL of the 0.1 mM group was higher (*p* < 0.05) than that of the other groups.

**Table 2:** Effect of different concentrations of ALA on spermatozoa kinetic parameters.

Parameter	Time	0 mM	0.025 mM	0.05 mM	0.1 mM	0.5 mM	1 mM
VSL (μm/s)	1 d	44.42 ± 0.14^Ad^	46.48 ± 0.20^Abc^	48.02 ± 0.46^Aa^	48.60 ± 0.26^Aa^	46.98 ± 0.31^Ab^	45.67 ± 0.37^Ac^
	3 d	43.65 ± 0.12^ABc^	44.69 ± 0.53^Bbc^	45.40 ± 0.46^Bab^	46.37 ± 0.18^Ba^	44.58 ± 0.09^Bbc^	44.78 ± 0.39^Ab^
	5 d	42.47 ± 0.67^Bb^	42.85 ± 0.32^Cb^	42.56 ± 0.22^Cb^	44.98 ± 0.26^Ca^	43.13 ± 0.44^Cb^	42.59 ± 0.06^Bb^
VCL (μm/s)	1 d	79.91 ± 0.49^Ac^	80.98 ± 0.37^Ab^	81.33 ± 0.10^Ab^	83.55 ± 0.15^Aa^	83.54 ± 0.34^Aa^	84.08 ± 0.44^Aa^
	3 d	76.03 ± 0.17^Bc^	79.19 ± 0.08^Bb^	79.64 ± 0.07^Bb^	82.44 ± 0.42^Ba^	79.49 ± 0.62^Bb^	79.65 ± 0.56^Bb^
	5 d	69.34 ± 0.06^Cd^	76.24 ± 0.30^Cb^	77.37 ± 0.13^Ca^	78.01 ± 0.21^Ca^	78.19 ± 0.37^Ba^	73.78 ± 0.61^Cc^
VAP (μm/s)	1 d	56.74 ± 0.36^Ab^	57.94 ± 0.52^Ab^	58.21 ± 0.31^Ab^	62.57 ± 0.33^Aa^	62.29 ± 0.28^Aa^	62.54 ± 0.94^Aa^
	3 d	54.77 ± 0.09^Bd^	56.15 ± 0.14^Bc^	57.08 ± 0.38^Ab^	59.34 ± 0.24^Ba^	59.65 ± 0.26^Ba^	58.85 ± 0.44^Ba^
	5 d	52.25 ± 0.16^Cd^	53.41 ± 0.12^Cc^	55.04 ± 0.42^Bb^	57.73 ± 0.15^Ca^	57.81 ± 0.52^Ca^	54.35 ± 0.15^Cb^
ALH (μm)	1 d	23.6 ± 0.16^Ac^	24.76 ± 0.08^Ab^	24.34 ± 0.09^Ab^	26.41 ± 0.37^Aa^	25.8 ± 0.12^Aa^	25.81 ± 0.26^Aa^
	3 d	22.5 ± 0.17^Bd^	23.19 ± 0.1^Bcd^	23.64 ± 0.16^Bbc^	24.88 ± 0.26^Ba^	24.33 ± 0.17^Bab^	23.92 ± 0.47^Bbc^
	5 d	20.73 ± 0.41^Cc^	22.08 ± 0.01^Cb^	22.37 ± 0.21^Cb^	23.65 ± 0.22^Ca^	23.57 ± 0.24^Ca^	22.51 ± 0.06^Cb^

Different superscripts (lowercase) in the same row show significant differences (*p* < 0.05). Different superscripts (uppercase) in the same column show significant differences (*p* < 0.05).

As indicated in Table 2, the VCL of the ALA groups was significantly higher (*p* < 0.05) than that of the control group from day 1 to 5. Specifically, on the first day, the VCL of the 0.1 mM, 0.5 mM and 1 mM groups was significantly higher (*p* < 0.05) than that of the other groups. On the third day, the VCL of the 0.1 mM group was significantly higher (*p* < 0.05) compared to the other groups. On the fifth day, the VCL of the 0.05 mM, 0.1 mM and 0.5 mM groups were significantly higher (*p* < 0.05) than those of the other groups.

As indicated in Table 2, the VAP of the 0.1 mM, 0.5 mM and 1 mM groups was significantly higher (*p* < 0.05) than that of the other groups from day 1 to 3. On the fifth day, the VAP of the 0.1 mM and 0.5 mM groups was significantly higher (*p* < 0.05) than that of the other groups.

Results showed that the ALH of the 0.1 mM, 0.5 mM and 1 mM groups was significantly higher (*p* < 0.05) than that of the other groups on the first day. Additionally, there were no significant differences (*p* > 0.05) in ALH between these three groups. On the third and fifth days, the ALH of the 0.1 mM group was significantly higher (*p* < 0.05) than that of the 0 mM, 0.025 mM, 0.05 mM and 1 mM groups, but it was not significantly higher (*p* > 0.05) than the 0.5 mM group.

### 3.3 Effects of ALA supplementation on spermatozoa plasma and acrosome membrane integrity

The plasma membrane integrity of the 0.05 mM and 0.1 mM groups was higher (*p* < 0.05) than that of the other groups on the first day. Additionally, these two groups were not significantly (*p* > 0.05) different from each other on the first day as shown in Table 3. The integrity of the plasma membrane in the 0.1 mM group was significantly higher (*p* < 0.05) than in the other groups from day 3 to 5.

**Table 3:** Effect of different concentrations of ALA on spermatozoa plasma and acrosome membrane integrity.

Parameter	Time	0 mM	0.025 mM	0.05 mM	0.1 mM	0.5 mM	1 mM
Plasma membrane (%)	1d	76.53 ± 0.63^Ad^	77.35 ± 0.15^Acd^	81.27 ± 0.36^Aa^	82.64 ± 0.81^Aa^	78.65 ± 0.23^Abc^	79.51 ± 0.20^Ab^
	3d	68.69 ± 0.51^Bd^	70.65 ± 0.28^Bc^	72.17 ± 0.43^Bb^	73.79 ± 0.32^Ba^	72.25 ± 0.49^Bb^	70.39 ± 0.39^Bc^
	5d	57.45 ± 1.06^Cc^	59.20 ± 0.24^Cc^	61.01 ± 0.43^Cb^	64.15 ± 0.53^Ca^	57.96 ± 0.24^Cc^	57.98 ± 0.28^Cc^
Acrosome integrity (%)	1d	77.17 ± 0.28^Ab^	78.06 ± 0.08^Ab^	79.93 ± 0.09^Aa^	80.12 ± 0.12^Aa^	78.09 ± 0.47^Ab^	78.08 ± 0.88^Ab^
	3d	71.58 ± 0.33^Bc^	71.98 ± 0.24^Bc^	73.77 ± 0.24^Bb^	75.53 ± 0.83^Ba^	71.52 ± 0.08^Bc^	71.24 ± 0.42^Bc^
	5d	66.24 ± 0.11^Ce^	69.53 ± 0.18^Cbc^	69.78 ± 0.13^Cb^	72.56 ± 0.25^Ca^	69.23 ± 0.12^Cc^	68.58 ± 0.06^Cd^

Different superscripts (lowercase) in the same row show significant differences (*p* < 0.05). Different superscripts (uppercase) in the same column show significant differences (*p* < 0.05).

On the first day of preservation, the integrity of the acrosome membrane in the 0.05 mM and 0.1 mM groups was significantly higher (*p* < 0.05) compared to the other groups on the first day as indicated in Table 3. The integrity of the acrosome membrane in the 0.1 mM group was significantly higher (*p* < 0.05) than in the other groups from day 3 to 5 and the acrosome membrane integrity of the control group was significantly lower (*p* < 0.05) than that of the ALA groups on the fifth day.

### 3.4 Effects of ALA supplementation on spermatozoa ROS content

**Figure 2:**
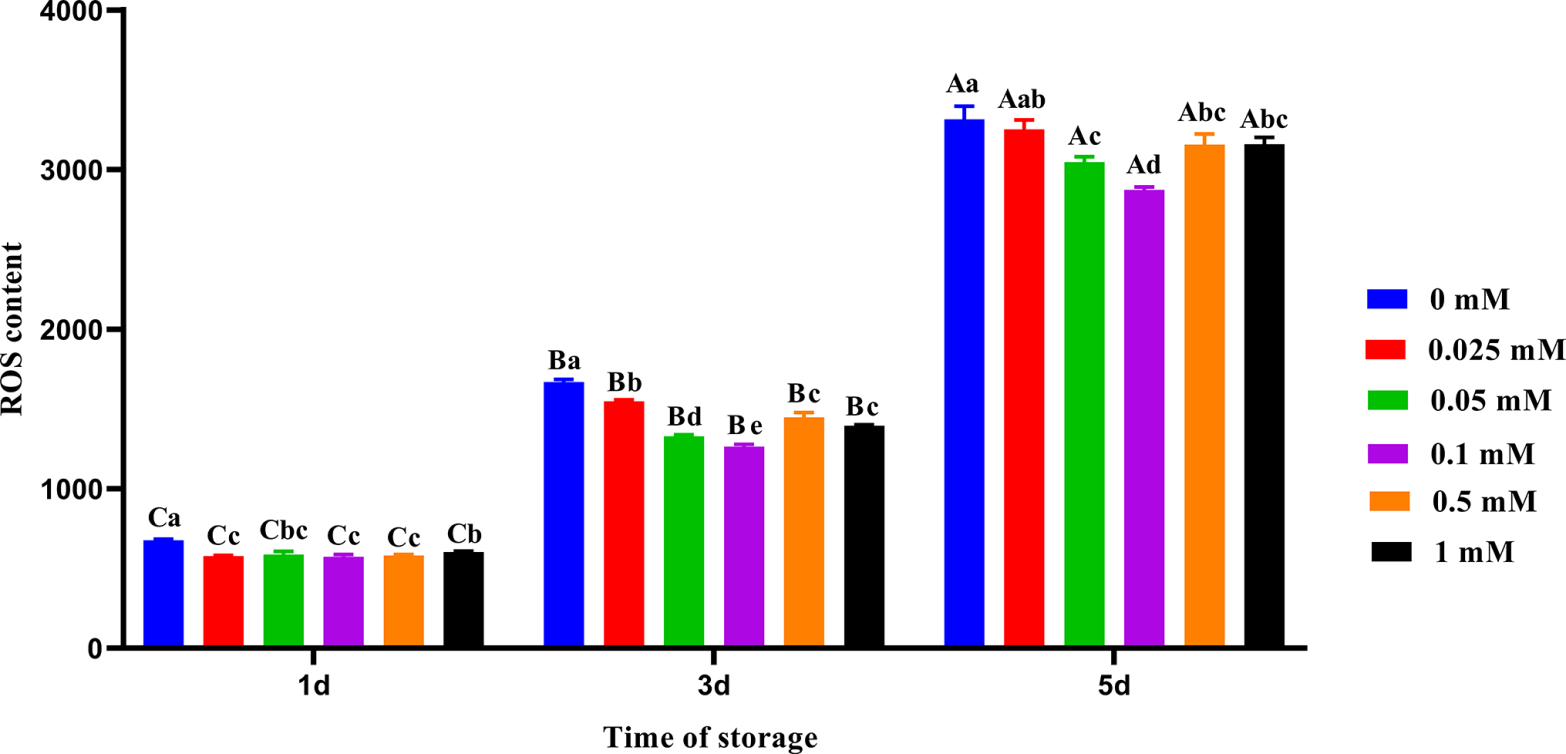
Effect of different concentrations of ALA on the ROS level of chilled spermatozoa from day 1 to 5. Values with different lowercase letters indicate difference (*p* < 0.05) among groups at each time point. Values with different uppercase letters indicate difference (*p* < 0.05) over time within the groups.

As shown in Figure 2, the ROS level in the control group was significantly higher (*p* < 0.05) than that in the group with ALA on the first and third days. The addition of ALA can reduce the ROS content in spermatozoa, and the ROS content in the 0.1 mM ALA group was the lowest from day 1 to 5. On the fifth day, the ROS level decreased initially with the increase of ALA concentration in each ALA addition group, and then increased. The ROS level in the 0.1 mM group was significantly lower (*p* < 0.05) than that of the other groups, and the inhibitory effect of the 0.1 mM group on the ROS level was more pronounced.

### 3.5 Effects of ALA supplementation on spermatozoa MDA content

**Figure 3:**
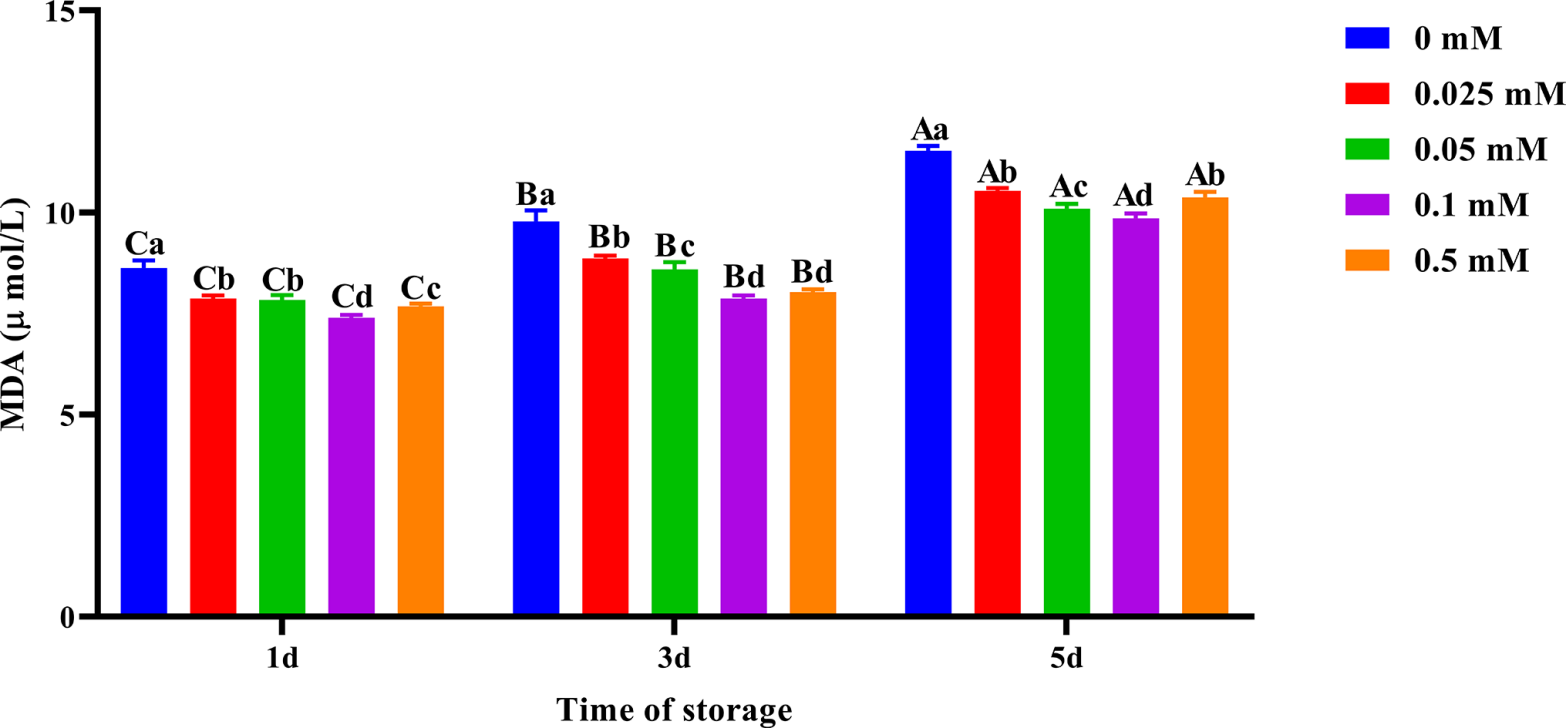
Effect of different concentrations of ALA on MDA content of chilled spermatozoa from day 1 to 5. Values with different lowercase letters indicate difference (*p* < 0.05) among groups at each time point. Values with different uppercase letters indicate difference (*p* < 0.05) over time within the groups.

On the first, third and fifth days, the spermatozoa MDA content in the ALA group was significantly lower (*p* < 0.05) than that of the control group as shown in Figure 3. MDA production gradually decreased with increasing concentration on the third and fifth days. However, when the concentration of ALA exceeded 0.1 mM, MDA production began to increase once more. On the fifth day of preservation, the spermatozoa MDA content of the 0.1 mM group was significantly lower (*p* < 0.05) than that of the other groups.

### 3.6 Effects of ALA supplementation on spermatozoa SOD activity

**Figure 4:**
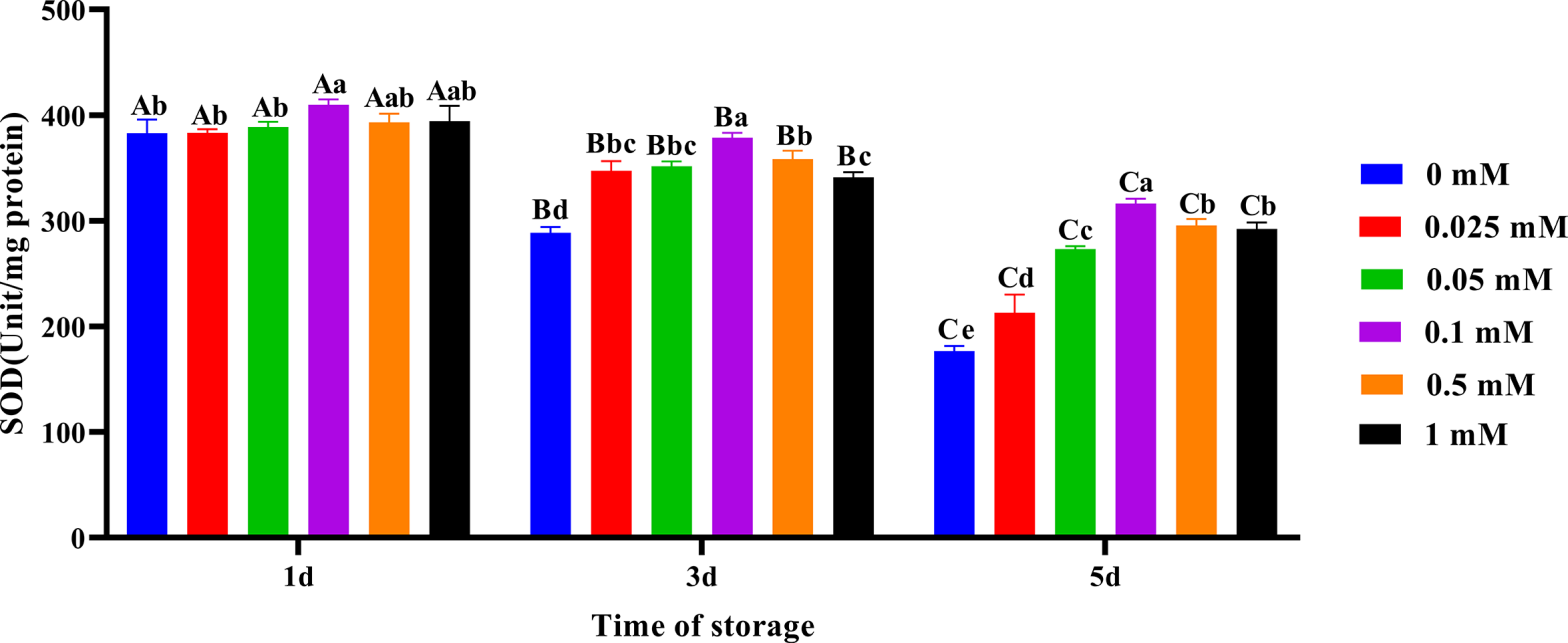
Effect of different concentrations of ALA on SOD activity of chilled spermatozoa from day 1 to 5. Values with different lowercase letters indicate difference (*p* < 0.05) among groups at each time point. Values with different uppercase letters indicate difference (*p* < 0.05) over time within the groups.

As shown in Figure 4, on the first day of preservation, the spermatozoa SOD activity in the 0.1 mM group was the highest and significantly higher (*p* < 0.05) than in the 0, 0.025 mM and 0.05 mM groups. On the third and fifth days, the spermatozoa SOD activity in the ALA addition group was significantly higher (*p* < 0.05) than that in the control group. Compared to other groups of ALA supplementation, the spermatozoa SOD activity was higher in the 0.1 mM group.

### 3.7 Effects of ALA supplementation on semen TAC

**Figure 5:**
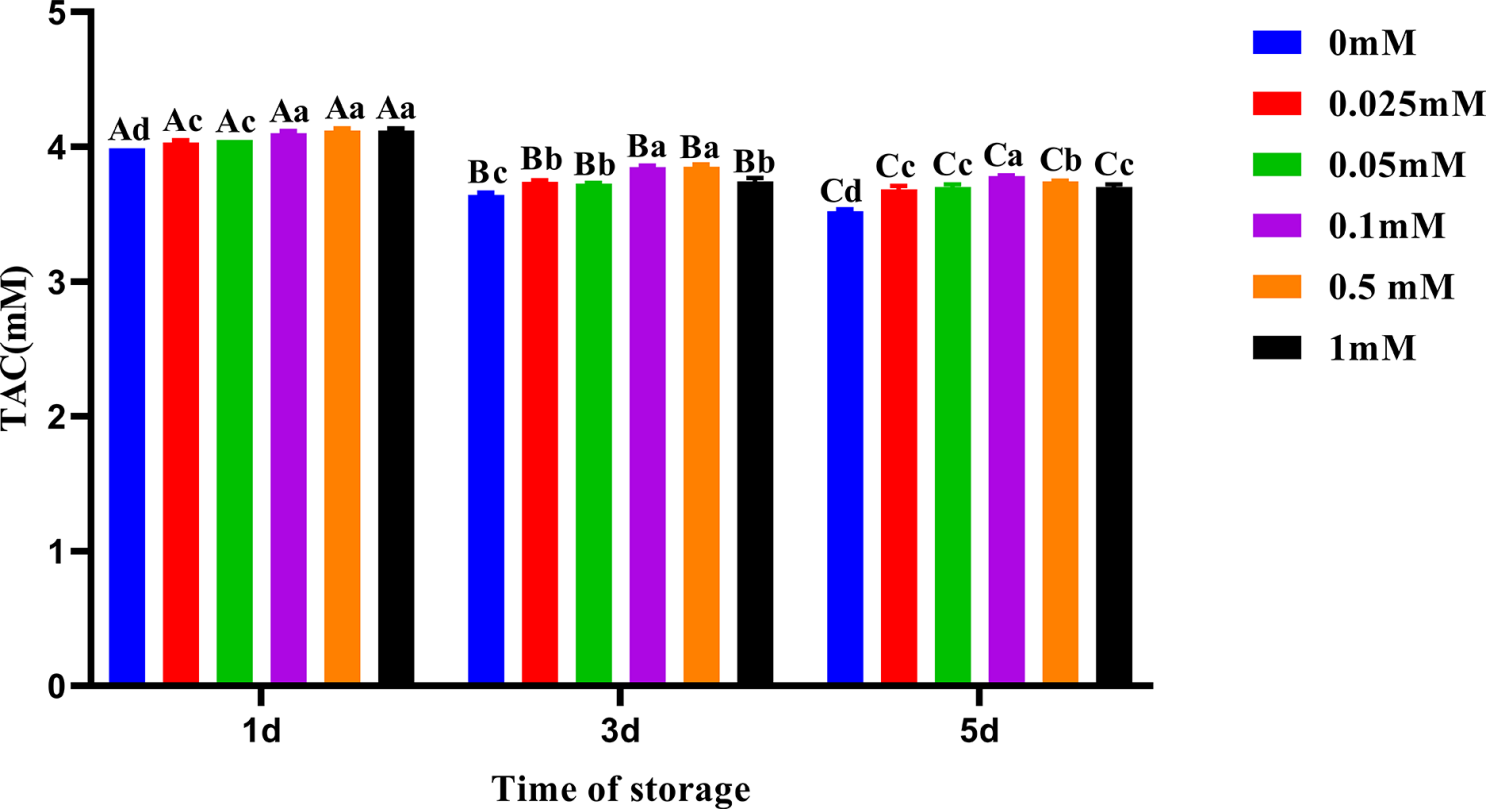
Effect of different concentrations of ALA on TAC of chilled spermatozoa from day 1 to 5. Values with different lowercase letters indicate difference (*p* < 0.05) among groups at each time point. Values with different uppercase letters indicate difference (*p* < 0.05) over time within the groups.

Compared to other groups, the decline in semen TAC in the 0.1 mM group was the slowest as shown in Figure 5. On the first, third, and fifth days, the TAC of the group with added ALA was significantly higher (*p* < 0.05) than that of the control group. On the first day, the semen TAC in the 0.1 mM, 0.5 mM and 1 mM groups was higher than that in the other three groups (*p* < 0.05). On the third day, the TAC of the 0.1 mM and 0.5 mM groups was significantly higher (*p* < 0.05) than that in the other four groups. On the fifth day, the TAC of the 0.1 mM group was significantly higher (*p* < 0.05) than that of other groups.

### 3.8 Effects of ALA supplementation on spermatozoa mitochondrial membrane potential

**Figure 6:**
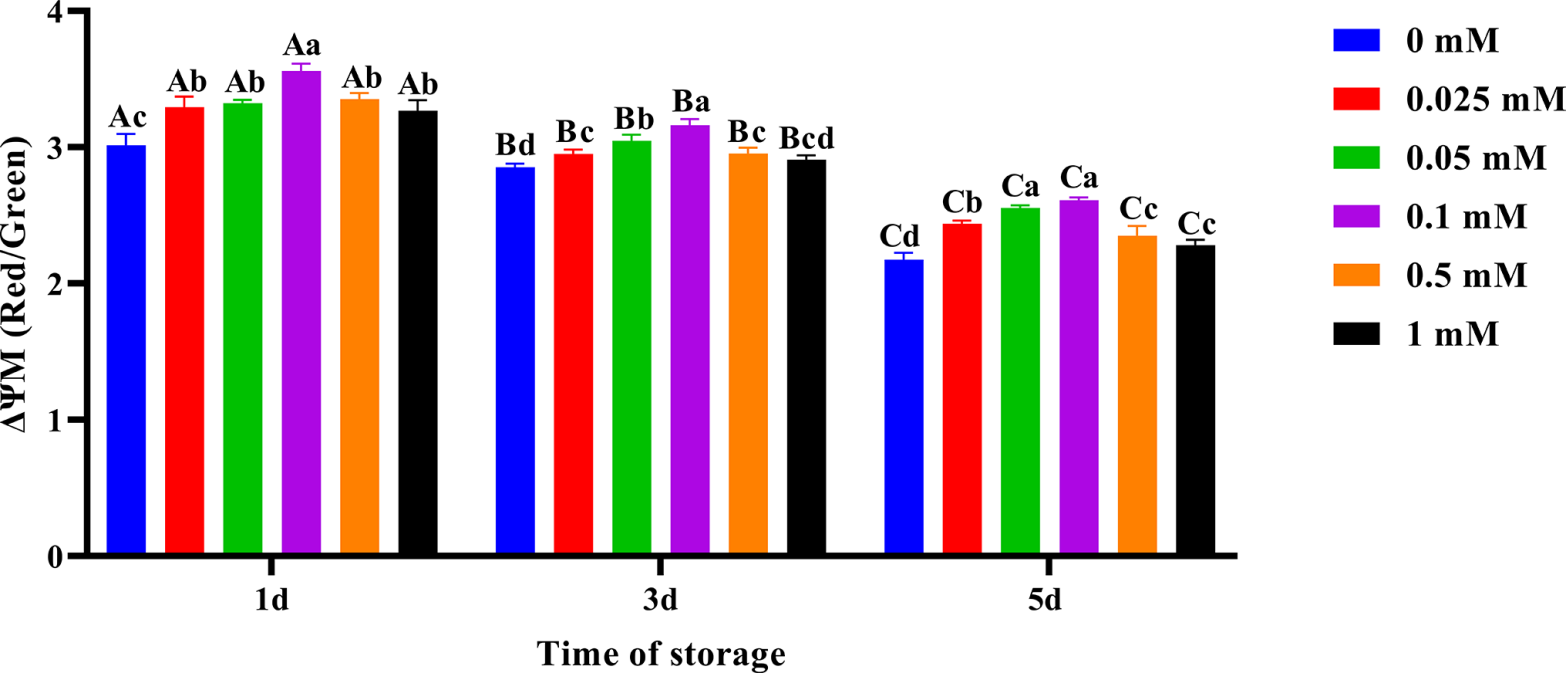
Effect of different concentrations of ALA on the mitochondrial membrane potential of chilled spermatozoa from day 1 to 5. Values with different lowercase letters indicate difference (*p* < 0.05) among groups at each time point. Values with different uppercase letters indicate difference (*p* < 0.05) over time within the groups.

As shown in Figure 6, the decrease in spermatozoa ΔΨM in the ALA addition group was slower compared to the control group, and the spermatozoa ΔΨM in the 0.1 mM group was higher. On the first day of preservation, the spermatozoa ΔΨM in the 0.1 mM group was significantly higher (*p* < 0.05) than that of the other groups. Meanwhile, the spermatozoa ΔΨM in the control group was significantly lower (*p* < 0.05) than that of the groups with added ALA. On the third day, the spermatozoa ΔΨM in the 0.1 mM group was significantly higher (*p* < 0.05) than that of the other groups. On the fifth day of preservation, the spermatozoa ΔΨM in the 0.05 mM and 0.1 mM groups was significantly higher (*p* < 0.05) than that of the other groups.

### 3.9 Effects of ALA supplementation on spermatozoa ATP content

**Figure 7:**
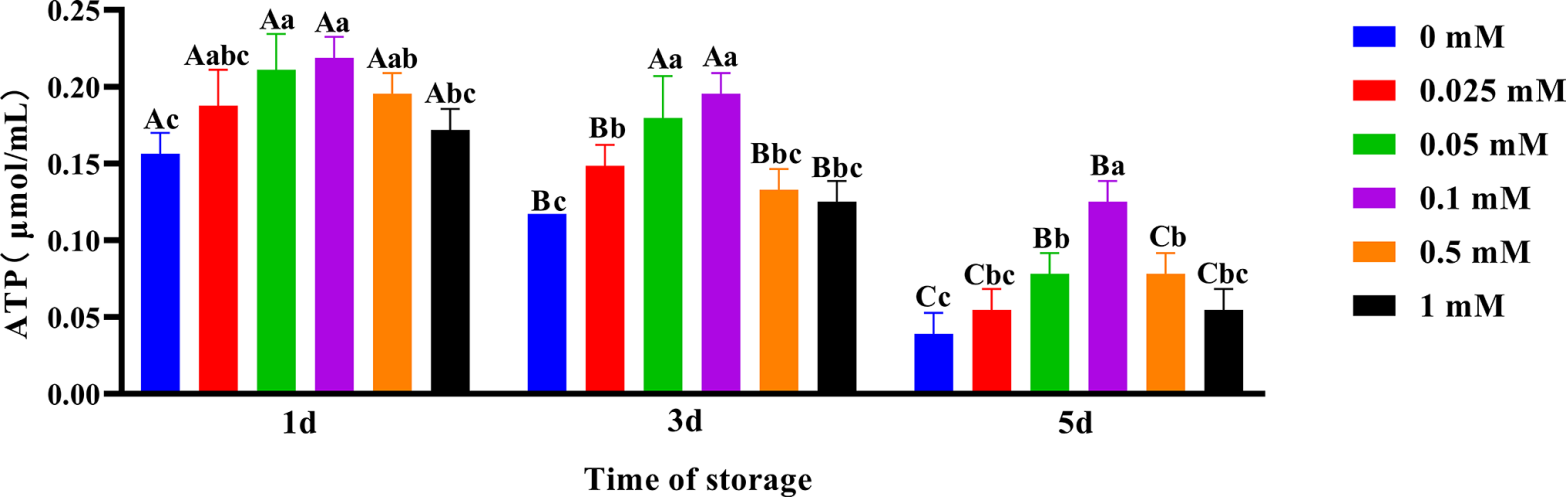
Effect of different concentrations of ALA on ATP content of chilled spermatozoa from day 1 to 5. Values with different lowercase letters indicate difference (*p* < 0.05) among groups at each time point. Values with different uppercase letters indicate difference (*p* < 0.05) over time within the groups.

As shown in Figure 7, the spermatozoa ATP content in the ALA addition group slowly decreased compared to the control group. On the first day, the spermatozoa ATP content in the 0.1 mM group was the highest. On the third day, the spermatozoa ATP content in the 0.05 mM and 0.1 mM groups was significantly higher (*p* < 0.05) than that of other groups. On the fifth day, the spermatozoa ATP content in the 0.1 mM group was significantly higher (*p* < 0.05) than that of the other groups.

## 4 Discussion

Spermatozoa are prone to the accumulation of ROS during liquid storage at 4°C, which can lead to the destruction of the spermatozoa structural integrity (25, 26). The quality of semen will deteriorate further with prolonged storage time, ultimately leading to a loss of spermatozoa function and fertilization ability. There are two ways for spermatozoa to produce ROS at 4°C. First, it is produced by the electron transport chain of spermatozoa mitochondria (27). Second, it is produced by the NADPH-dependent oxidase system on the spermatozoa plasma membrane (28). Therefore, controlling the production of ROS and LPO is crucial for reducing oxidative damage to spermatozoa. Moreover, ALA is a nucleophilic reagent containing a mercaptan group, which can react with endogenous electrophilic reagents (29). Therefore, it can effectively remove ROS during semen preservation and has a good scavenging effect on free radicals, such as hydroxyl free radicals, nitric oxide free radicals, and peroxynitrite (30).

TM and PM are the most direct and important parameters of sperm quality. VSL, VCL, VAP, and ALH are important parameters for evaluating sperm kinematic parameters, and fertilization ability is closely related to these kinematic parameters. In this study, the addition of exogenous ALA significantly improved spermatozoa TM, PM and kinetic parameters compared to the control group. This may be due to the high concentration of ROS in semen affecting the parameters of spermatozoa motility. This study demonstrated that the semen diluent containing 0.1 mM ALA resulted in lower ROS content on the third and fifth days. It has been demonstrated that 0.1 mM ALA exhibits a strong ability to scavenge ROS. Furthermore, it can effectively reduce the oxidative damage to spermatozoa caused by ROS and maintain optimal spermatozoa motility parameters. Kasimanickam et al. (31) observed that the spermatozoa SOD activity had a positive effect on the spermatozoa kinetic parameters during liquid storage at 4°C. The spermatozoa SOD activity in the ALA group was significantly higher than that of the control group from day 3 to 5. Additionally, the spermatozoa VCL, VAP, and ALH were also significantly higher than those of the control group on the fifth day in this study. Ibrahim et al. (32) reported that a concentration of 0.02 mmol/mL ALA significantly improved the motility of Boer bucks’ spermatozoa after thawing. In a study on cryopreserved boar spermatozoa, Shen et al. (33) reported that a concentration of 6 mg/mL ALA was found to be the most effective in significantly increasing spermatozoa motility after thawing. These results are consistent with the findings of this study, indicating that ALA can enhance spermatozoa quality. However, it is possible that the concentration of ALA varies among different species and under different preservation conditions.

Spermatozoa plasma membrane contains a lot of unsaturated fatty acids, which are prone to oxidation and can result in sperm damage (25, 26). At the same time, ROS produced by spermatozoa metabolism can also lead to peroxidation damage to the spermatozoa structure of Hu ram. Because ALA is both fat-soluble and water-soluble. Therefore, it will combine with a phospholipid bilayer and liquid components that envelop the spermatozoa when it is absorbed by the spermatozoa membrane (34). Finally, a barrier is formed on the spermatozoa membrane to enhance the spermatozoa tolerance to free radical attack and ultimately ensure the spermatozoa structural integrity (35). In this study, the integrity rate of the plasma membrane and spermatozoa acrosome in the ALA group was significantly higher than that in the control group, indicating that ALA had a protective effect on the structural integrity of the spermatozoa membrane. Onder et al. (36) reported that ALA could enhance the plasma membrane integrity of ram spermatozoa after thawing. Avdatek and Gündoğan (37) found that the addition of 1 mM ALA had a postive protective effect on the structural integrity of cryopreserved goat spermatozoa. These studies were consistent with the findings of our study. MDA is the product of LPO, which can disrupt the membrane structure and ultimately impact spermatozoa function. Excessive ROS can lead to damage to the integrity of the spermatozoa plasma membrane, its fluidity, and enzyme receptor function (38). This study found that the exogenous addition of ALA provided a layer of protection to the spermatozoa membrane, inhibited LPO, and significantly reduced the content of MDA in semen (39). These findings indicate that ALA had a protective effect on the spermatozoa plasma membrane and acrosome. This may be the cause of the decrease in MDA.

Spermatozoa motility depends on the supply of energy. Oxidative decarboxylation with ALA will impact the concentration of cytochrome c and alter the mitochondrial membrane potential (40). Fayyaz et al. (41) research demonstrated that an optimal concentration of ALA can regulate mitochondrial coenzyme metabolism and utilization efficiency, maintain a high mitochondrial membrane potential, and ultimately protect mitochondrial function. This study found that compared to the control group, the addition of suitable ALA could maintain a higher level of mitochondrial membrane potential in spermatozoa, which was beneficial for spermatozoa motility. However, the protective effect of a high concentration of ALA is diminished, and the mitochondrial membrane potential was reduced. This may be due to a higher concentration of antioxidants causing some damage to spermatozoa mitochondria (42). In all ALA treatment groups, the addition of 0.05 mM and 0.1 mM ALA can result in higher mitochondrial membrane potential in spermatozoa on the fifth day of preservation. Therefore, an optimal concentration of ALA may reduce oxidative damage by ultimately Dihydrolipoic Acid (DHLA), maintaining high mitochondrial membrane potential to protecte mitochondrial function, and ultimately enhancing semen quality. This guarantees the implementation of AI in production. Considering the results of the present study, future research should assess how ALA exerts beneficial effects on the 4°C preservation of Hu ram spermatozoa and conduct IVF experiments.

## 5 Conclusion

The results of the work revealed the protective effects of ALA on Hu ram spermatozoa membrane functionality and motility during storage at 4°C. In addition, supplementation with ALA to semen decreased the contents of MDA and ROS, increased the SOD activity, the ATP content and spermatozoa ΔΨM and TAC, and ultimately improved the motility of Hu ram spermatozoa preserved at 4°C. The optimal concentration of ALA was determined to be 0.1 mM.

## Data availability statement

The original contributions presented in the study are included in the article/supplementary material, further inquiries can be directed to the corresponding author.

## Ethics statement

The animal studies were approved by Animal Ethics Committee of Yangzhou University. The studies were conducted in accordance with the local legislation and institutional requirements. Written informed consent was obtained from the owners for the participation of their animals in this study.

## Author contributions

XS: Writing – review & editing. LZ: Writing – original draft, Software. YK: Methodology, Investigation, Writing – original draft. XW: Data curation, Writing – review & editing. CJ: Software, Writing – review & editing. JW: Resources, Writing – review & editing. TS: Visualization, Writing – review & editing. YL: Project administration, Supervision, Writing – review & editing.
